# Detection of chikungunya virus DNA using two-dimensional MoS_2_ nanosheets based disposable biosensor

**DOI:** 10.1038/s41598-018-25824-8

**Published:** 2018-05-16

**Authors:** Chaitali Singhal, Manika Khanuja, Nahid Chaudhary, C. S. Pundir, Jagriti Narang

**Affiliations:** 1 Amity Institute of Nanotechnology, Amity University, Noida, UP India; 2 Centre for Nanoscience and Nanotechnology, Jamia Millia Islamia, New Delhi, 110025 India; 3 Department of Biochemistry, Maharishi Dayanand University, Rohtak, Haryana India

## Abstract

Development of platforms for a reliable, rapid, sensitive and selective detection of chikungunya virus (CHIGV) is the need of the hour in developing countries. To the best of our knowledge, there are no reports available for the electrochemical detection of CHIGVDNA. Therefore, we aim at developing a biosensor based on molybdenum disulphide nanosheets (MoS_2_ NSs) for the point-of-care diagnosis of CHIGV. Briefly, MoS_2_ NSs were synthesized by chemical route and characterized using scanning electron microscopy, transmission electron microscopy, UV-Vis spectroscopy, Raman spectroscopy and X-Ray Diffraction. MoS_2_ NSs were then subjected to physical adsorption onto the screen printed gold electrodes (SPGEs) and then employed for the detection of CHIGV DNA using electrochemical voltammetric techniques. Herein, the role of MoS_2_ NSs is to provide biocompatibility to the biological recognition element on the surface of the screen printed electrodes. The detection strategy employed herein is the ability of methylene blue to interact differentially with the guanine bases of the single and double-stranded DNA which leads to change in the magnitude of the voltammetric signal. The proposed genosensor exhibited a wide linear range of 0.1 nM to 100 µM towards the chikungunya virus DNA.

## Introduction

Chikungunya (CHIG) is a disease caused by chikungunya virus (CHIGV); which is a RNA virus belonging to *Alphavirus* genus and *Togaviridae* family^1^. CHIG was discovered in Tanzania (1952) and since then four types of its genotypes have been identified so far^2^. These include East-Central South African (ECSA), West African, Asian, and the Indian Ocean Lineage (IOL)^3,4^. This disease transmits to the humans by infected mosquitoes, namely; *Aedes agypti* and *Aedes albopictis*^5^. CHIG has been characterized by onset of sudden fever followed by skin rashes, rigorous joint pain and relentless rheumatic symptoms^1,6^. The acute infection of CHIG is self-limiting and the symptoms mostly resolve within weeks to years^7^. Though rarely fatal; CHIG has emerged as a major public health concern recently; due to its enormous outbreaks all over the world^8^. According to World Health Organization, there were 6,93,489 suspected and 37,480 confirmed cases of chikungunya as reported by Pan American Health Organization (PAHO) regional office^1^. The massive outbreak in India in 2016 has left long lasting effects on the population^9^. Looking at the rate at which CHIG is spreading, its rapid and early diagnosis is the most significant challenge for government aided health care agencies and developing countries.

Detection of CHIG RNA through RT-PCR from serum samples or determination of serum antibodies (IgM) are the diagnostic measures followed conventionally^9^. These methods are time consuming and the procedure is cumbersome. Thus, rapid and early monitoring point-of-care (POC) diagnostic tool has become the need of hour. Advancements in electrochemical biosensors have motivated various designs of real time POC diagnosis tools^10^. The advantages such as rapid response time, low cost and suitability for mass production associated with detection of DNA hybridization have triggered the development of DNA-based electrochemical biosensors^5,11,12^. These advantages motivated the present work wherein we have developed an electrochemical DNA biosensor for the detection of CHIGV DNA. A practical advantage of electrochemical detection could have future implications in translating to cheap assays using single-use screen-printed electrodes (SPEs), which is an ideal tool due to their low cost, disposability and design flexibility as compared to traditional electrode materials^13–15^. Hence, SPEs serve as a transition away from the traditional cumbersome beaker-type electrochemical cells and bulky electrodes^16^.

Previously several DNA biosensors have been reported involving labeling of PCR products with enzymes^17^, redox active components^18^ or nanoparticles^19,20^ to enhance the electrochemical signal. Nanomaterials have been used as carrier beacons for indirect; however, vigorous and precise means for detecting target molecules. Two-dimensional (2D) molybdenum disulphide (MoS_2_) nanomaterials belonging to transition-metal dichalcogenides have been gaining much attention these days^21^. This is because MoS_2_ has emerged as a material with exceptional biocompatibility, good electrochemical catalysts activity, easy modification^22^, high specific surface area and large junction area of the electrode/electrolyte^23^ and sensitive surface states (high surface-to-volume ratio)^24^. Each Mo is coordinated to six S atoms; by stacking covalently bound S–Mo–S via weak van der Waals interactions^25^, thereby enhancing the planar electric transportation properties^26,27^. MoS_2_ nanosheets (MoS_2_ NSs) are capable enough to adsorb single-stranded DNA by the van der Waals force between nucleobases and the basal plane of MoS_2_NSs^24,25^. These advantages along with the existence of suitable bandgap in comparison to graphene and graphene oxides which have small or no band gaps makes MoS_2_NSs highly suitable for sensors that can detect DNA, proteins, metal ions, and other compounds.

In the present report, an electrochemical DNA biosensor has been prepared for the detection of DNA of chikungunya virus electrochemically. Screen printed disposable gold electrodes coated with molybdenum disulphide nanosheets have been used as the platform for immobilization of the probe DNA and employed for the detection of the target DNA.

## Results and Discussion

### Assay design and principle

**Figure 1:**
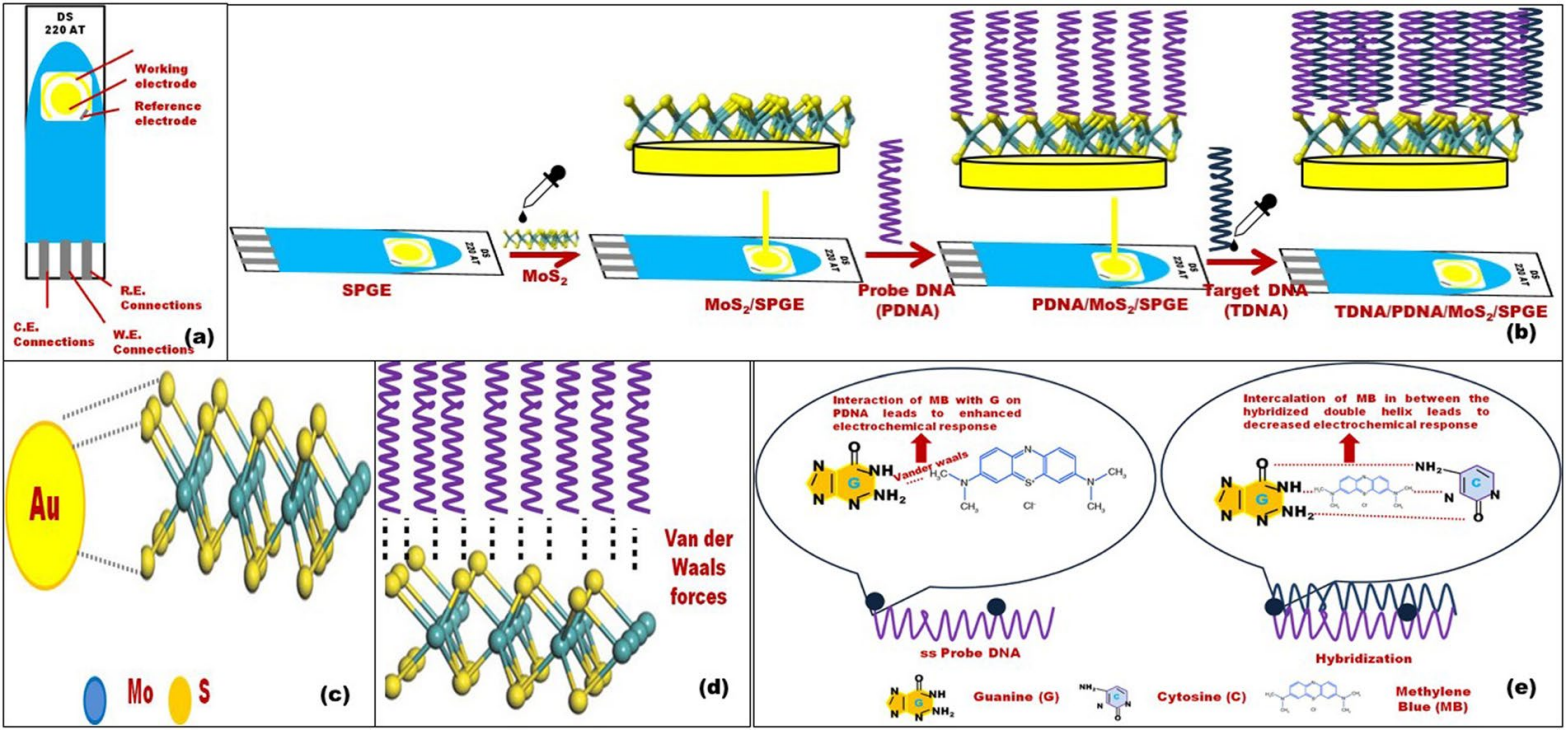
(**a**) Screen printed gold electrode (SPGE). (**b**) Stepwise representation of the fabrication of SPGE with MoS_2_ nanosheets, probe DNA and target DNA. (**c**) Interaction of working electrode (Au) of SPGE and MoS_2_ shows strong affinity between Au (SPGE) and S (MoS_2_). (**d**) MoS_2_ interacts with probe DNA via Vander Waals forces. (**e**) Principle for the detection of hybridization of target DNA via redox hybridization indicator, i.e., methylene blue.

A schematic representation showing the main steps in our assay for detection of target CHIG DNA and the principle behind the detection is represented in Fig. 1. Figure 1(a) shows the various components of SPGE. The working electrode (WE) and counter electrode (CE) were made of gold and the reference electrode (RE) was made of silver. The main steps involved in the fabrication of SPGEs were presented in Fig. 1(b). The gold on the WE of SPGE shows strong affinity for the sulfur group in MoS_2_ (Fig. 1(c)). This confirms a strong immobilization of MoS_2_ over SPGE. Probe DNA of CHIGV was immobilized over MoS_2_ coated SPGE (Fig. 1(b)). The MoS_2_ are capable enough to adsorb single-stranded DNA by the van der Waals force between nucleobases^24,25^ and the basal plane of MoS_2_NSs (Fig. 1(d)). Thereby ensuring efficient immobilization. Further, the target DNA was added (Fig. 1(b)) along with MB and the hybridization was allowed to occur for 60 sec. The role of MB has been presented schematically in Fig. 1(e). The interaction of MB with the free guanine bases of the single-stranded DNA leads to enhanced electrochemical response. This is due to its ability to attract to guanine via Vander Waals interaction. Upon hybridization, the MB gets intercalated between the bulky double helix of the double-stranded DNA. Thus, a decreased response was observed as shown in Fig. 1(e).

### Characterization of the synthesized MoS_2_ nanosheets (MoS_2_ NSs)

**Figure 2:**
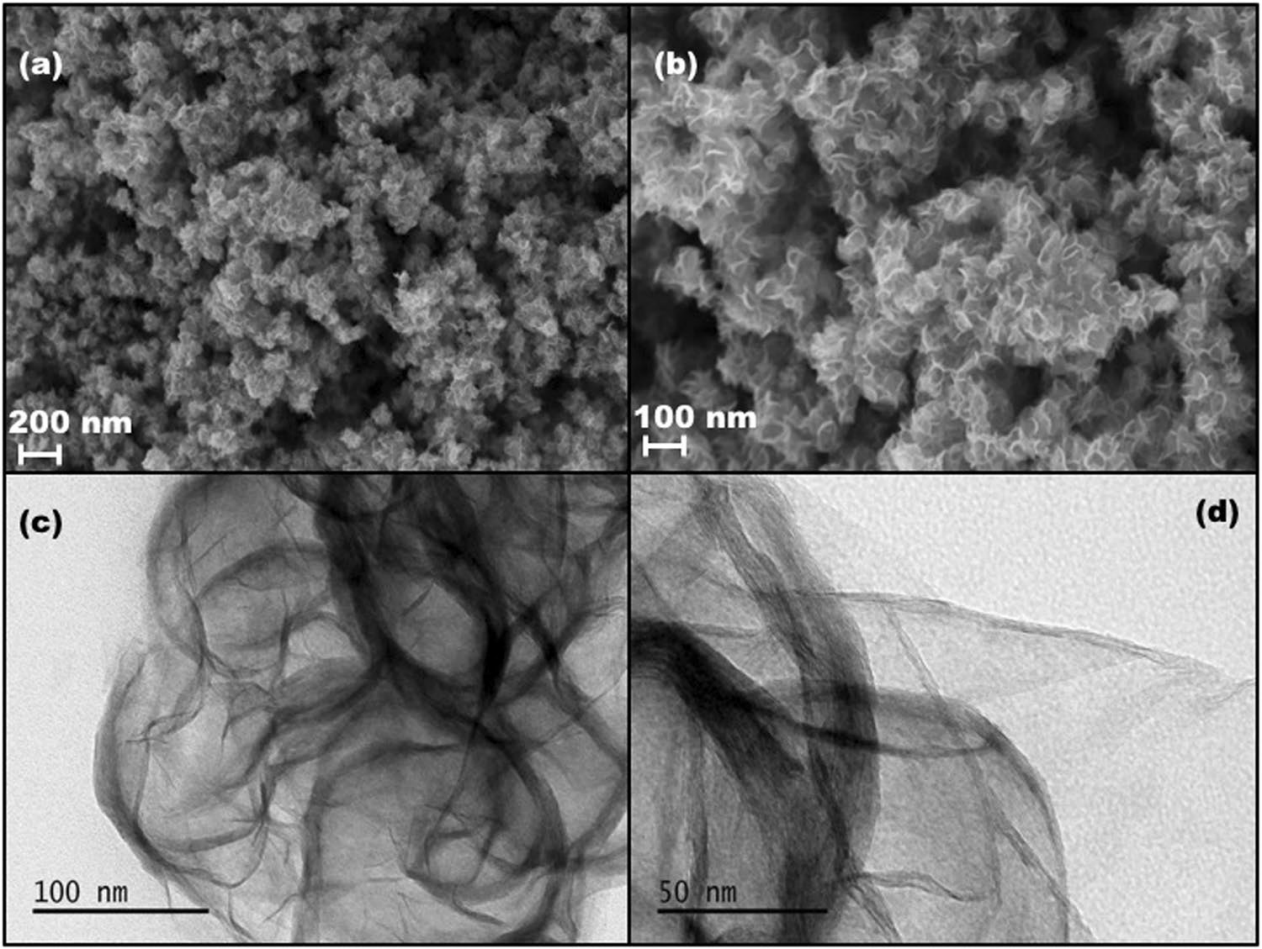
(**a**) Scanning electron micrograph of MoS_2_ nanosheets at 200 nm. (**b**) Scanning electron micrograph of MoS_2_ nanosheets at 100 nm. (**c**) Transmission electron micrograph of MoS_2_ nanosheets at 100 nm. (**d**) Transmission electron micrograph of MoS_2_ nanosheets at 50 nm.

SEM images clearly showed that highly dense, laminar nanosheets with curved edges. The nanosheets are folded at the edges giving them a petal like shape (Fig. 2(a,b)). Transmission electron microscopy is an excellent tool to characterize two dimensional transition metal dichalcogenides (TMDs). The MoS_2_ monolayer is composed of three atom layers: Mo layer sandwiched between two sulphur layers. The three layers are stacked via weak vander Waal interaction. TEM micrographs (Fig. 2(c,d)) clearly showed that thin layer structures of MoS_2_ nanosheets. The interplanar spacing is found to be 0.64 nm which is in agreement with the earlier reports^28^.

**Figure 3:**
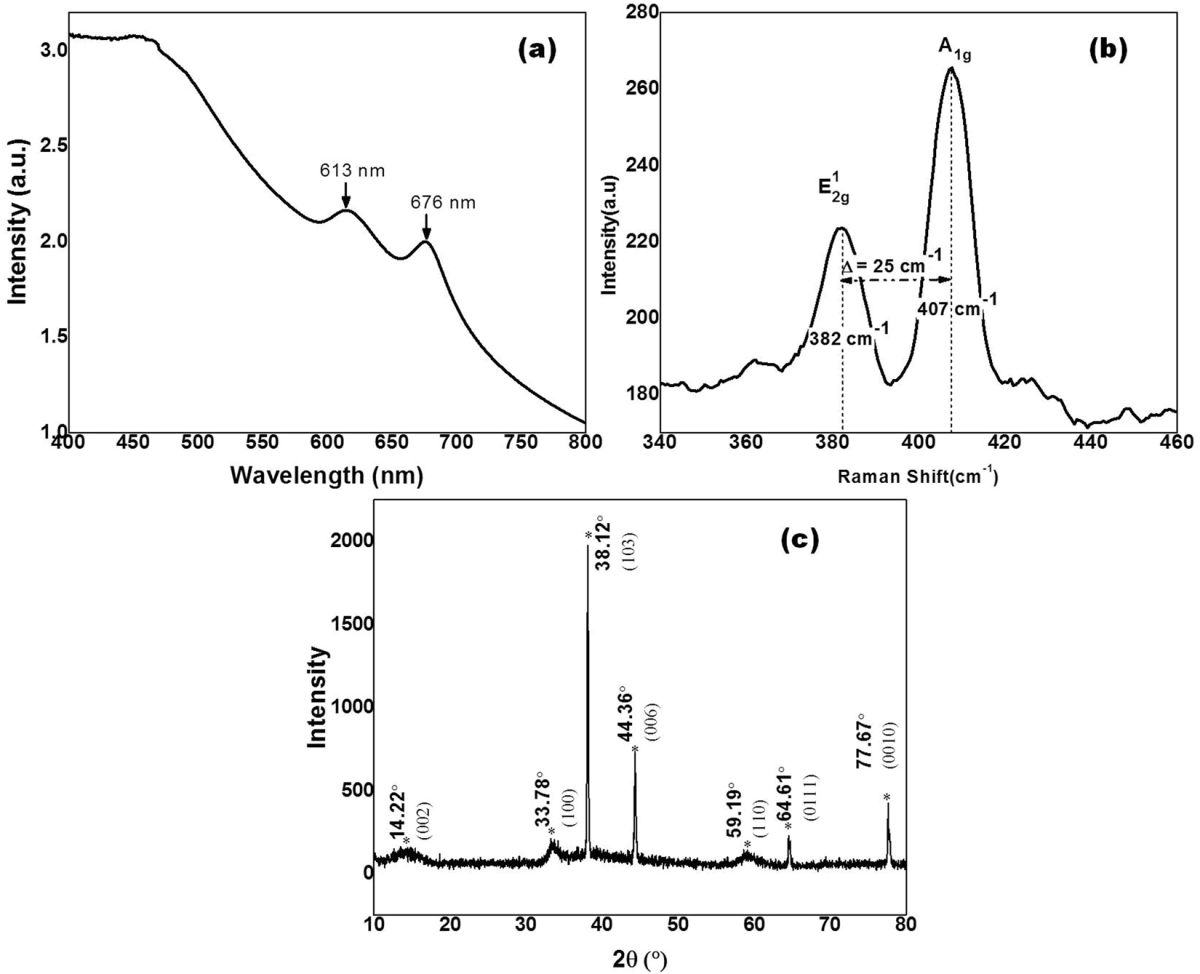
(**a**) UV-Vis spectroscopy of MoS_2_ NSs. (**b**) Raman spectra of MoS_2_ NSs. (**c**) X-Ray Diffraction spectra of MoS_2_ NSs.

Figure 3(a) shows the UV-Vis absorption spectra of MoS_2_ nanosheets. Two characteristic absorption peaks (A and B) were observed at 676 nm (1.83 eV) and 66 613 nm (2.02 eV). These exciton peaks correspond to A and B direct electronic transition of MoS_2_ nanosheets, originated from the energy split of valence band and spin orbit coupling^29^.

Figure 3(b) shows the Raman spectrum of as synthesized MoS_2_ nanosheets. A typical two pronounced peaks were observed at 382 cm^−1^ and 407 cm^−1^. The Raman peak at 382 cm^−1^ (E^1^_2g_) is associated with the in-plane MoS_2_ phonon mode and 407 cm^−1^ (A_1g_) is due to the out of plane MoS_2_ phonon mode. The difference between these characteristic peaks is 25 cm^−1^ implying that nanosheets consist of 5 or more MoS_2_ layers is stacked together. These two characteristic peaks indicate that synthesized MoS_2_ nanosheets possess 2H-MoS_2_ structure^30^. The crystal structure of the MoS_2_ nanosheets was investigated through X-ray diffraction as shown in the Fig. 3(c). All the diffraction peaks can be indexed reported MoS_2_ phase (JCPDS card No. 37–1492). The peaks at 14.22°, 33.78°, 38.12°, 44.36°, 59.19°, 64.61° and 77.67° can be ascribed to (002), (100), (103), (006), (110), (0111) and (0010) planes of MoS_2_, respectively.

### Optimization of the concentration of the MoS_2_ nanosheets and PDNA concentration

**Figure 4:**
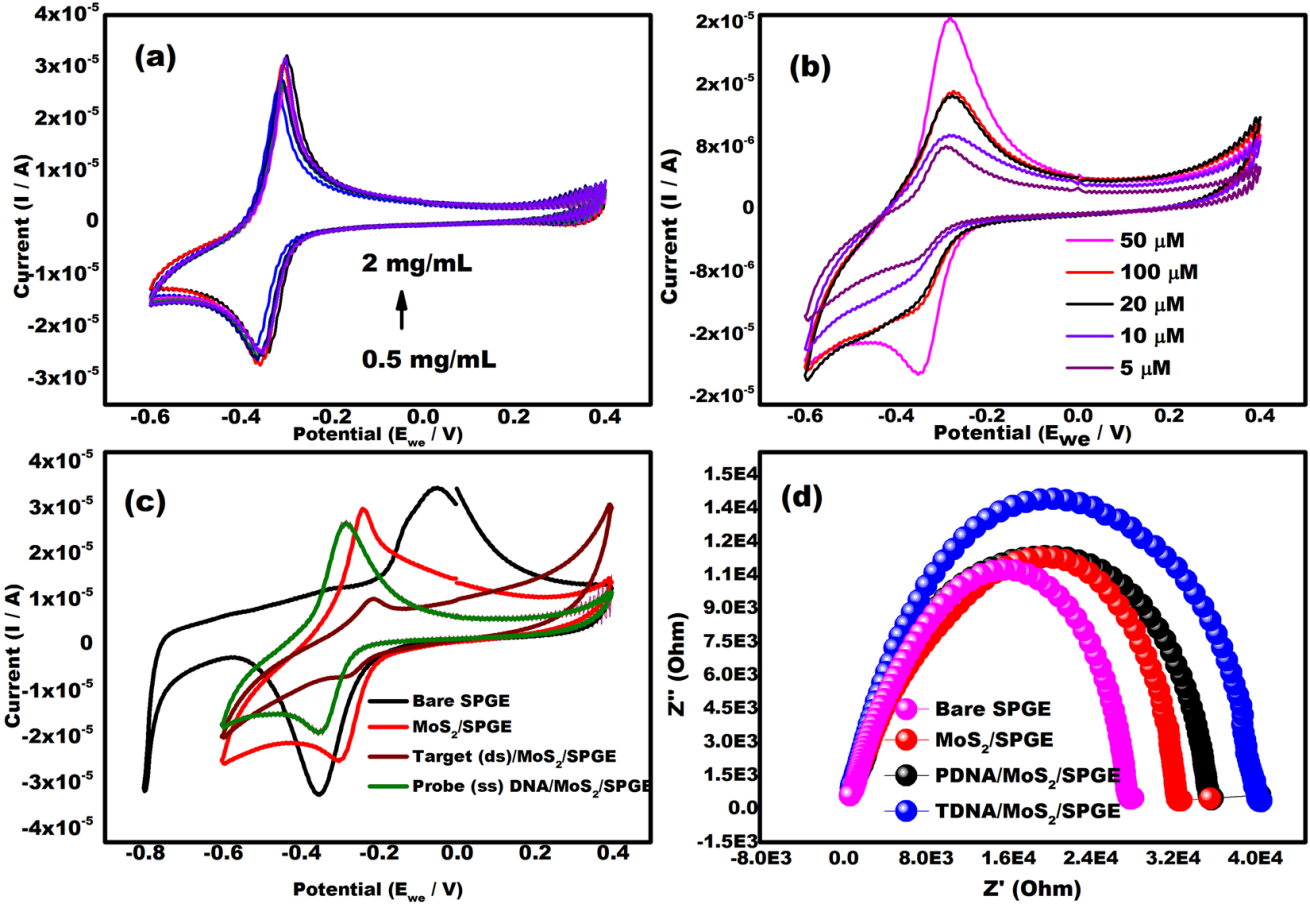
(**a**) Electrochemical ability of different concentrations of MoS_2_ nanosheets deposited on SPGEs from a concentration of 0.5 to 2 mg/mL in 0.1 M PBS containing 1 µM MB at the scan rate of 100 mVs^−1^ in the potential range from −0.6 to +0.4 V. (**b**) Cyclic voltammograms of PDNA/MoS_2_NSs/SPGEs in 0.1 M PBS containing 1 µM MB at various concentrations of probe DNA ranging from 5 µM to 100 µM at the scan rate of 100 mVs^−1^ in the potential range from −0.6 to +0.4 V. (**c**) Cyclic voltammograms at various stages of screen printed gold electrode (SPGE) including bare SPGE, MoS_2_ coated SPGE, probe DNA coated SPGE and target DNA coated SPGE in 0.1 M phosphate buffer saline (pH 7.8), 1 µM MB in the potential range from −0.6 to +0.4 V. (**d**) Nyquist plot at various stages of screen printed gold electrode (SPGE) including bare SPGE, MoS_2_ coated SPGE, probe DNA coated SPGE and target DNA coated SPGE in 0.1 M phosphate buffer saline (pH 7.8) and 1 µM MB (frequency range of 100 Hz–10^3^ kHz).

Optimization of the concentration of MoS_2_ nanosheets and probe DNA is an important parameter to ensure sufficient hybridization. The cyclic voltammograms of various concentrations of MoS_2_ and PDNA were shown in Fig. 4(a,b) respectively. The concentration of MoS_2_ was varied from 0.5 mg/mL to 2 mg/mL and the electrochemical response was observed. The highest response occurred at 1 mg/mL (Fig. 4(a)). Above 1 mg/mL, the response was stable so this was chosen as the concentration of MoS_2_ for the future experiments.

Figure 4(b) shows that the highest peak current was observed when PDNA concentration was 50 µM. At 100 µM PDNA, the signal drastically reduced. This was due to the increased thickness of the organic layer at the surface of SPGE which decreased the electron transfer rates. Consequently, 50 µM was chosen as the most favorable concentration of PDNA.

### Electrochemical analysis at various stages of the SPGEs

The electrochemical behaviors of various stages of SPGEs obtained after modification with MoS_2_NSs, PDNA or TDNA were analyzed using CV in 0.1 M phosphate buffer saline (pH 7.8) and 1 µM MB in the potential range from −0.6 to +0.4 V at a scan rate of 100 mVs^−1^ (Fig. 4(c)). All modified SPGEs exhibited a pair of well-defined redox peaks due to the oxidation and reduction of methylene blue at SPGE. As can be seen in Fig. 4(c), bare SPGE shows the maximum electrochemical response of 3.2 × 10^−5^ A (Ia) and −3.5 × 10^−5^ A (Ic) due to the presence of gold which shows good metallic conductivity. MoS_2_ NSs shows decreased current response of 2.9 × 10^−5^ A (Ia) and −2.8 × 10^−5^ A (Ic) due to the semi-conducting nature of MoS_2_ in comparison to the conducting gold (bare SPGE). In spite of the decreased electrochemical response, the MoS_2_ nanosheets are preferred; due to their ability to adsorb ssDNA by the van der Waals force between nucleobases and the basal plane of MoS_2_NSs^24,25^. The immobilized probe DNA shows further decrease in the current response to 2.5 × 10^−5^ A (Ia) and −2.2 × 10^−5^ A (Ic). This is due to the insulating nature of the DNA. As stated above, hybridization of the target DNA with the probe DNA further decreases the electrochemical response to 6 × 10^−6^ A (Ia) and −8 × 10^−6^ A (Ic).

The voltage for the peaks of methylene blue were −0.22 V (Ea) −0.29 V (Ec) at MoS_2_NSs modified SPEs and shifted to more negative values (Ea = −0.31 V, Ec = −0.38 V) when analyzed with the probe DNA modified SPE, reverting to more positive potentials (Ea = −0.19 V, Ec = −0.28 V) upon hybridization of the chikungunya DNA.

The explanation for this behavior is explained by Raveendran *et al*.^31^. As per their report, the shift in the potential over the surface of SPGEs lies on the negatively charged nature of DNA and the transfer of electrons during the hybridization event. MoS_2_NSs transfer the electrons from the MB and vice versa during cyclic voltammetry to produce the characteristic voltammogram at the specified voltage. Modification of MoS_2_NSs by the probe DNA modifies this transfer of electrons reducing the potential at which this is occurring, making it easier for MB to reduce. During the hybridization of the CHIGV target DNA with the complementary strand of the probe DNA immobilized on the surface there is a restructuration of the molecules and a higher demand for electrons, which leads to reduction of MB with less available electrons for the molecule, hence increasing the reduction potential.

The similar response was observed in Fig. 4(d) which shows Nyquist plot at various stages of SPGE. The resistance charge transfer (Rct) value of bare SPGE was lowest and that of target DNA was highest. The probe DNA show increased Rct value in comparison to MoS_2_ NSs. Since, the resistance is inversely proportional to the current; therefore, the Nyquist plot and CV results were in line with one another.

### Electrochemical response of PDNA/MoS_2_NSs/SPGEs at various scan rates

**Figure 5:**
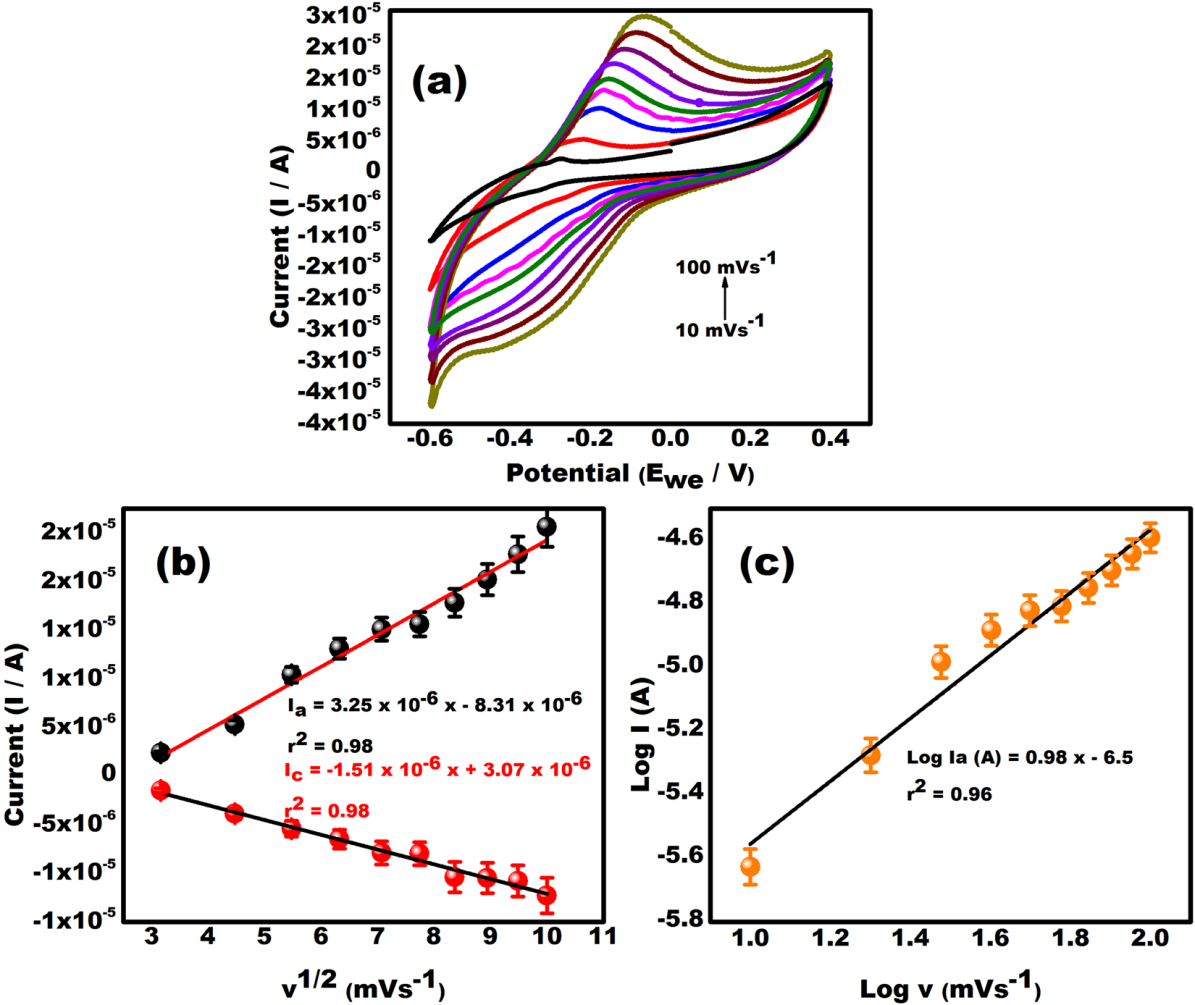
(**a**) Cyclic Voltammograms obtained at PDNA/MoS_2_ NSs/SPGE for scan rates ranging from 10–100 mVs^−1^ in 0.1 M phosphate buffer saline (pH 7.8) and 1 µM MB in the potential range from −0.6 to +0.4 V. (**b**) The dependency of peak currents on the square root of potential sweep rates in a wide range of 10–100 mVs^−1^ (**c**) Dependence of log of peak current on log v (mVs^−1^).

The effect of scan rates ranging from 10 to 100 mVs^−1^ on the PDNA/MoS_2_NSs/SPGEs is shown in Fig. 5(a). It shows that the anodic and cathodic peak current of MB on the PDNA biosensor increased constantly from 10 to 100 mVs^−1^, confirming that the electrochemical reaction process of the biosensor is mainly diffusion controlled. However, to assure the stability of the biosensor response, 100 mVs^−1^ was chosen as the scan rate for subsequent studies.

The oxidation (Ia) and reduction peak current (Ip) both were proportional to square root of scan rate (v^1/2^) in Fig. 5(b) which is expressed as Ia = 3.25 × 10^−6^ × −8.31 × 10^−6^, r^2^ = 0.98, Ic = −1.51 × 10^−6^ × +3.07 × 10^−6^, r^2^ = 0.98. This makes it clear that the process of catalysis is diffusion controlled rather than surface controlled under the condition of sufficient potential^32,33^. The diffusion controlled behavior of the reaction is confirmed by a plot between Log Ia and Log v as; Log Ia = 0.98 log v (mVs^−1^) − 6.5, r^2^ = 0.96 as shown in Fig. 5(c).

### Analytical performance of the biosensor

**Figure 6:**
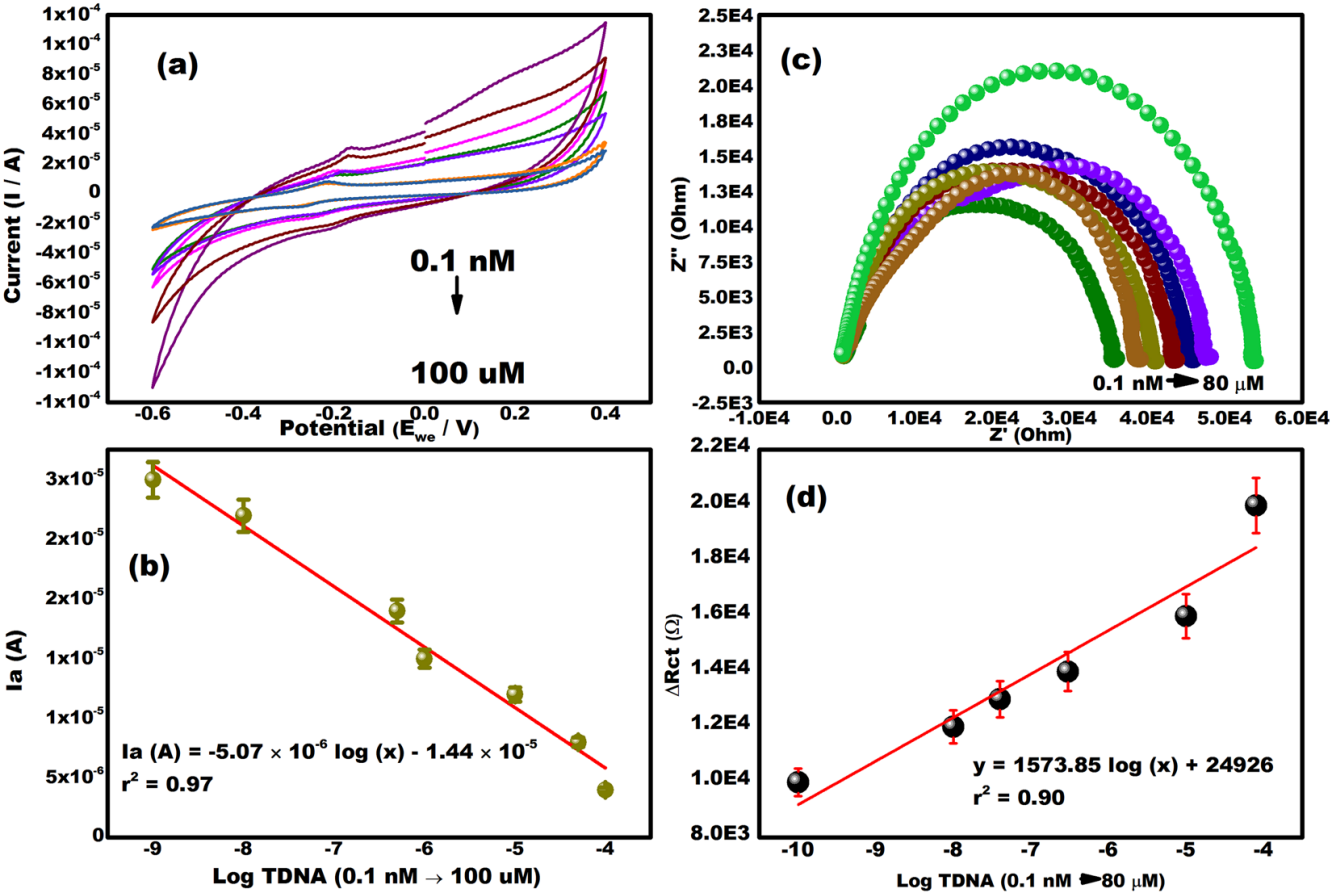
(**a**) Cyclic Voltammograms verifying hybridization of the different conc. of the complementary target DNA at PDNA/MoS_2_NSs/SPGE (0.1 nM to 100 µM) in 0.1 M phosphate buffer saline (pH 7.8) and 1 µM MB in the potential range from −0.6 to +0.4 V. (**b**) Nyquist Plot verifying hybridization of the different conc. of the complementary target DNA at PDNA/MoS_2_NSs/SPGE (0.1 nM to 80 µM) in 0.1 M phosphate buffer saline (pH 7.8) and 1 µM MB (frequency range of 100 Hz–10^3^ kHz). (**c**) The calibration plot of the TDNA/PDNA/MoS_2_NSs/SPGE electrode as a function of the logarithmic concentration of the Target DNA and anodic peak current. (**d**) The calibration plot of the TDNA/PDNA/MoS_2_NSs/SPGE electrode as a function of the logarithmic concentration of the Target DNA and change in resistance charge transfer.

A close perusal of cyclic voltammograms shows that the response signal of MB decreases with the increase of TDNA concentration in the range 0.1 nM to 100 µM (Fig. 6(a)). The peak current has decreased due to the formation of bulky hybrid complexes which hinder the interaction of MB molecules with the pure PDNA on the electrode. MB behaves as an anionic indicator with the ability to bind with the unpaired guanine bases in the DNA strands. Therefore, higher number of unpaired guanine residues results in higher interaction of MB molecules with the DNA strand and thus gives higher current response. The hybridization of the probe and target DNA lead to the reduction in the availability of the unpaired guanine because of its hydrogen bonding with cytosine in the complimentary target DNA. This results in lesser interaction of MB and thus, lower current response. Therefore, the current response decreases after hybridization of the TDNA because of lesser interaction of MB with unpaired guanine. The similar analyses have been reported earlier as well^34,35^. Figure 6(c) shows a linear variation in peak currents with the concentrations of target nucleotide. The linear fitted relation is given below:

$${\rm{Ia}}\,=-\,5.07\times {10}^{-6}\,\mathrm{log}\,\times -\,1.44\times {10}^{-5},{{\rm{r}}}^{2}=0.97$$

The limit of detection (LOD) was calculated to be 3.4 nM in a 3 σ rule and limit of quantification (LOQ) was calculated to be 104.81 nM in a 10 σ rule. Our proposed sensor offers a wide linear range (0.1 nM to 100 µM) and sufficiently low detection limit for CHIGV detection.

Electrochemical Impedance Spectroscopy (EIS) was also done in order to confirm the hybridization of the probe DNA with the various concentrations of target DNA (0.1 nM to 80 µM). Figure 6(b) shows the nyquist plot indicating the hybridization of the target DNA to the PDNA. Upon increasing the concentration of the target DNA from 0.1 nM to 80 µM, the Rct value increased. This is because the results of EIS and CV are always contrary from one another. Thus, verifying hybridization. Figure 6(d) shows a linear variation in change in Rct with the log of the concentrations of target nucleotide. The linear fitted relation is given below:

$${\rm{y}}=1573.85\ast \mathrm{log}x+24926,\,{{\rm{r}}}^{2}=0.90$$

### Effect of pH, temperature and hybridization time on the PDNA biosensor

**Figure 7:**
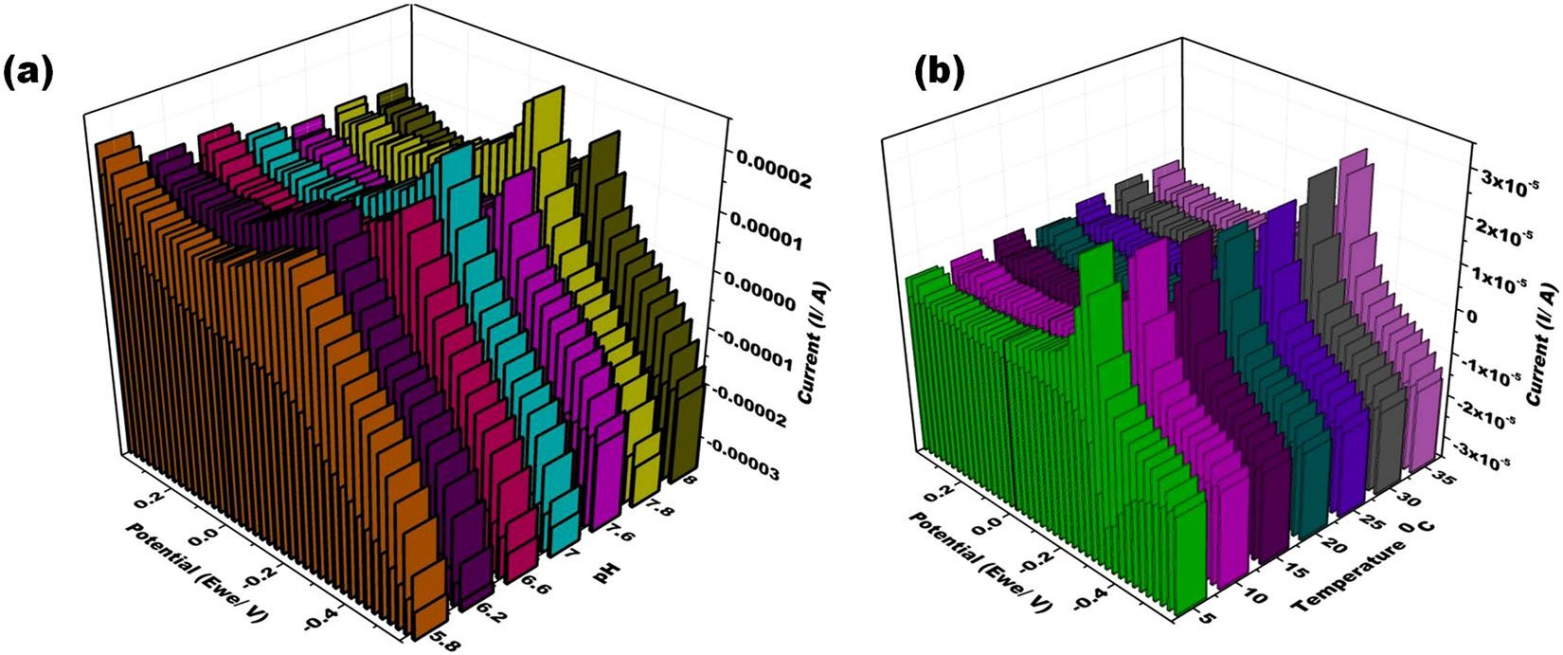
(**a**) 3D representation of the cyclic voltammogram at PDNA/MoS_2_NSs/SPGE for pH of 0.1 M phosphate buffer saline ranging from 5.8 to 8.0 each is having 1 µM MB in the potential range from −0.6 to +0.4 V at the scan rate of 100 mVs^−1^. (**b**) 3D representation of the cyclic voltammogram at PDNA/MoS_2_NSs/SPGE for temperatures ranging from 5 to 35 °C in 0.1 M phosphate buffer saline ranging having 1 µM MB in the potential range from −0.6 to +0.4 V at the scan rate of 100 mVs^−1^.

Figure 7(a) depicts the 3D representation of the cyclic voltammograms for the pH response of the biosensor. Since MoS_2_ is generally considered to be biocompatible with DNA, the pH response of the MB solution at PDNA/MoS_2_NSs/SPGE was studied. The alkaline conditions are beneficial for MB because MB (C_16_H_18_N_3_SCl) is a heterocyclic aromatic chemical compound and under alkaline conditions, it tends to form cations, whereas OH− is adsorbed to the surface of PDNA modified to form negatively charged adsorption centers, thus promoting the adsorption of MB ions^36^. The highest current response was observed at pH 7.8 after which the response became stable. Thus, pH 7.8 was chosen for the rest of the experiments.

The effect of temperature on the PDNA/MoS_2_NSs/SPGE was studied and the results are presented in Fig. 7(b)). As evident from the figure, the electrochemical response of the sensor enhanced upon increasing the temperature (though not much difference); because high temperatures are more favorable for hybridization (obvious from PCR). But in order to keep the simplicity of employment of the biosensor for general use, 35 °C was chosen as the temperature at which appropriate hybridization could take place.

The hybridization time is an important parameter in a DNA biosensor. Therefore, the hybridization time was optimized. For this, different electrodes with PDNA immobilized were prepared and TDNA was added with MB. Cyclic voltammetric measurements were recorded at various time intervals. After the analysis, 35 sec was kept as the optimum hybridization time for this biosensor.

### Real samples and selectivity analysis of the biosensor

**Figure 8:**
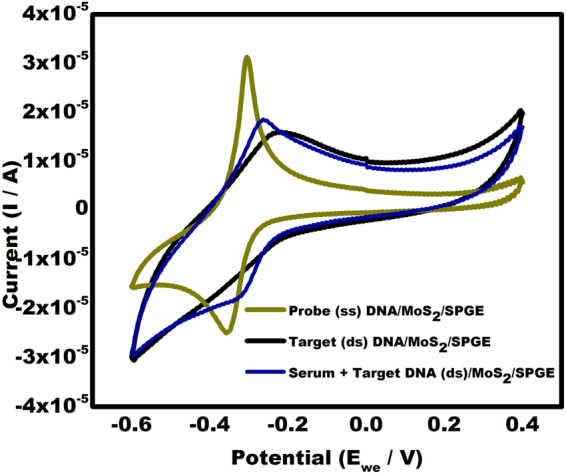
Cyclic voltammogram hybridization of chikungunya TDNA spiked in real sample (serum) in comparison with PDNA and TDNA in the potential range from −0.6 V to +0.4 V at a scan rate of 100 mVs^−1^.

The target DNA was spiked in purchased serum sample and dropped on the surface of the PDNA/MoS_2_NSs/SPGE. The hybridization of probe DNA with target DNA in serum sample occurred and the current response observed was similar to the current response obtained when TDNA was directly added over the PDNA/MoS_2_NSs/SPGE (Fig. 8). Thus, confirming the analysis in real samples.

**Figure 9:**
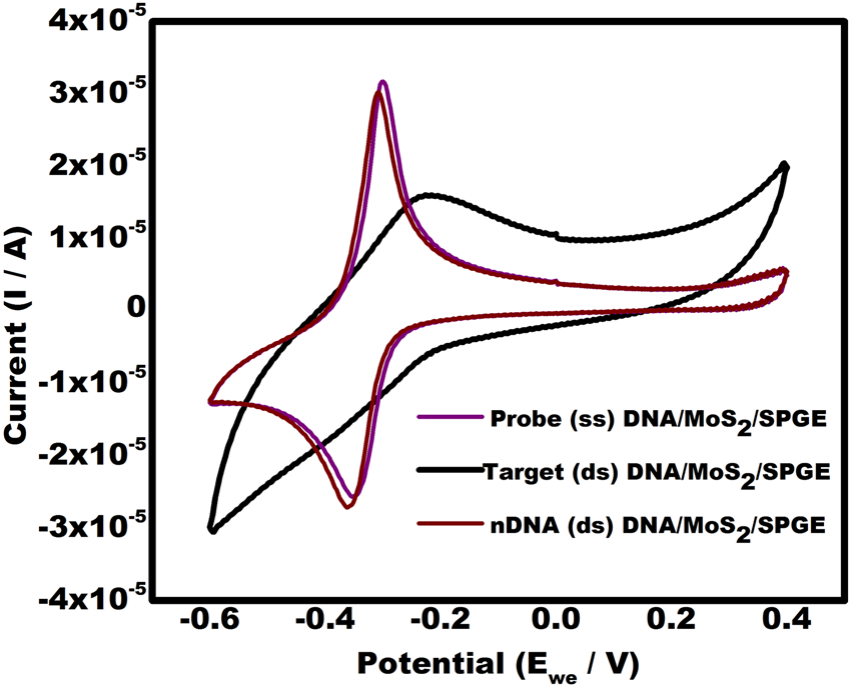
Cyclic voltammograms showing response of non-complementary DNA (nDNA) by the present sensor in comparison with PDNA and TDNA.

In order to investigate the selectivity of this biosensor, the current response obtained from PDNA/MoS_2_NSs/SPGE and hybridized TDNA/MoS_2_NSs/SPGE was compared to the non-complimentary DNA (n DNA). A significantly different current response was observed in case of nDNA when compared with the TDNA. The non-complimentary DNA showed response nearly equal to the PDNA (Fig. 9). Thus, no hybridization occurred with the nDNA and MB is free to interact with the guanine bases available in the ssDNA.

### Comparison study

The sensing interface ability of present genosensor was compared with earlier reported MoS_2_ based biosensors^37–52^. High sensitivity, specificity and repeatability of the sensor make it best among other biosensors (Supplementary Table 1).

## Methods

### Reagents and apparatus

Monosodium phosphate, Disodium phosphate was purchased from SRL, India. Methylene blue was purchased from Thermo Fischer Scientific, India. All the chemicals were of analytical reagent grade and used without further purification. Double-distilled water was used throughout this experiment. Tris-EDTA (TE buffer) buffer of pH 8.0 was used to prepare the 100 µM stock solutions of probe DNA and target DNA. Phosphate buffer saline (PBS, 0.1 M) was prepared by mixing the stock solutions of 1 M NaH_2_PO_4_ and Na_2_HPO_4_ and NaCl. 1 µM methylene blue (MB) prepared in PBS was used as buffer solution in all the electrochemical measurements. DRP-220 AT (screen printed with high temperature curing ink) screen printed gold electrodes (SPGEs) with 3.4 × 1.0 × 0.05 cm dimensions were purchased from DropSens (India). The SPGEs had working electrode (diameter of 1.4 mm) and counter electrodes made of gold whereas the reference electrode and the electrical contacts were made of silver. Human serum (minus IgA/IgM/IgG) was obtained as a lyophilized powder from Sigma Aldrich (India). Electrochemical measurements like cyclic voltammetry (CV) were measured on Autolab PGSTAT 204. Morphology of the MoS_2_ nanosheets was characterized by transmission electron microscope (TEM, FEI Tecnai G2, 300 KV) and scanning electron microscopy (JEOL JSM-6010LA). TEM sample preparation was done by placing a drop of MoS_2_ nanosheets on carbon coated copper grid followed by drying in air and transferred to the microscope operated at an accelerating voltage of 300 KV. The structure of MoS_2_ nanosheets were studied by X-Ray Diffraction. Sample was scanned in the range of 10 ° to 80 ° at a glancing angle of 1 °. The Raman spectrum was taken by using a Horiba micro-Raman confocal microscopic system (LabRAM), at room temperature in an ambient air. A spectrophotometer (Agilent Technologies, model no: Cary 100 series) was used to obtain the UV-Vis absorption spectra of the MoS_2_ nanosheets, which were recorded in the wavelength range of 300–800 nm at room temperature.

### Preparation of CHIG probe and target DNA

All the oligonucleotides were synthesized by Integrated DNA Technology (IDT) as lyophilized translucent films. The sequences were listed as follows:

Probe DNA: 5′-NH_2_- TGC TCC GCG TCC TTT ACC AA-3′

Target DNA: 5′-TTG GTA AAG GAC GCGGAG CA-3′

Non-complimentary (NC) DNA: 5′-CTA TGC TTA CAC GTA GAC TGT GC-3′.

### Synthesis of Molybdenum disulphide nanosheets (MoS_2_ NSs)

MoS_2_ NSs were synthesized by dissolving 3 mM of sodium molybdate dihydrate and 9 mM of thioacetamide in 50 mL of distilled water. Further 2.8 mM silicon tungstic acid was added into the reaction solution under violent stirring. The resultant solution was transferred into a 100 mL Teflon-lined stainless autoclave and was kept at 220 °C for 24 h. Then the autoclave was allowed to cool and the resulting products were filtered off, washed with 1 M NaOH, ethanol and distilled water for several times, and dried in vacuum at 50 °C for 8 h.

### Bioelectrode fabrication, immobilization of PDNA and hybridization of TDNA

The bare SPGEs were cleaned by washing them subsequently with gold cleaning solution and 0.1 M phosphate buffer saline. The synthesized MoS_2_ NSs (2.5 µL) were deposited physically on the surface of the working electrode (WE) of SPGEs. The drop-casted electrodes were left for drying at room temperature for 1.5 h. Since gold has a high affinity for sulfur, modification of SPGEs was made easy.

The MoS_2_ NSs/SPGEs were further interacted with the CHIG probe DNA. After drying, 3 µL of the probe DNA was deposited over the MoS_2_ NSs. The probe DNA immobilized MoS_2_ NSs/SPGEs were left for 3 h to allow complete immobilization. The probe DNA immobilized SPGE was further used for hybridization of the target DNA. The target DNA was added along with the hybridization indicator (MB) and after appropriate time (optimized); the electrochemical analysis was done to confirm hybridization.

### Optimization of pH, temperature and hybridization time

The probe DNA immobilized SPGE was optimized for hybridization at various pH (5.8 to 8) and temperature (20 to 40 °C). The pH, temperature showing best electrochemical response was finally used for hybridization with the target DNA. The hybridization time is also an important factor in a DNA biosensor and thus, the hybridization time between PDNA and TDNA was also optimized. Six electrodes having fixed concentration of probe DNA (50 µM) over MoS_2_ NSs were prepared and were incubated with complimentary target DNA (80 µM) for different intervals of time (10 to 60 s).

### Analysis of the stability, specificity and performance of the biosensor with real sample

The real sample analysis was done by adding known concentration of the target oligonucleotides in the purchased serum. This mixture along with PBS containing MB was dropped over probe DNA modified SPGE and sensing was done further. The stability analysis was performed by storing the probe DNA modified SPGE at 4 °C and periodically measuring the signal strength corresponding to it by CV and DPV upon addition of the target DNA. For selectivity analysis of the proposed DNA biosensor, the probe DNA modified SPGE was exposed to complementary and non-complementary target samples. The concentration of the non-complementary nucleotide sample was kept 3 orders higher than that of the complementary sample for determining the sensor selectivity. The probe modified electrodes was exposed to complementary and non-complementary target samples subsequently.

## Conclusions

A reliable electrochemical CHIGV DNA detection system has been developed in the present work. MoS_2_ nanosheets deposited screen printed gold electrodes proved efficient for probe DNA binding. The sensor shows good linear range from 0.1 nM to 100 µM with 3.4 nM as the limit of detection. Point of care technologies (POCTs) are the need of the hour. The features like less response time, high linearity and economic feasibility makes the present sensor beneficial to be miniaturized as POCTs.

## Electronic supplementary material

Supplementary Information
