# Supplementary material for: Detection of chikungunya virus DNA using two-dimensional MoS2 nanosheets based disposable biosensor
Source: Sci Rep. 2018 May 16;8:7734. doi: 10.1038/s41598-018-25824-8 (PMC5955964; doi:10.1038/s41598-018-25824-8)
Supplement: Supplementary file 1 — Supplementary Information [file 41598_2018_25824_MOESM1_ESM.docx]

**Detection of chikungunya virus DNA using two-dimensional MoS_2_ nanosheets based disposable biosensor**

**Chaitali Singhal^a^, Manika Khanuja^b^, Nahid Chaudhary^b^, C.S. Pundir^c#^, Jagriti Narang^a*^**

*^a^Amity Institute of Nanotechnology, Amity University, Noida (UP), India*

*^b^Centre for Nanoscience and Nanotechnology, Jamia Millia Islamia, New Delhi-110025, India*

*^c^Department of Biochemistry, Maharishi Dayanand University, Rohtak, (Haryana), India*

**^*^Corresponding author I**

**Dr. Jagriti Narang**

**Assistant Professor**

**AINT, Amity University, Noida (U.P)**

**Email: jags_biotech@yahoo.co.in**

**Telephone no. 9811792572**

**^#^Corresponding author II**

**Dr. Manika Khanuja**

**Assistant Professor**

**Centre for Nanoscience and Nanotechnology**

**Jamia Millia Islamia, New Delhi-110025, India**

**Email: manikakhanuja@gmail.com**

**Telephone no. 9810076796**

**^#^Corresponding author III**

**Prof. C.S. Pundir**

**Emeritus Scientist**

**Maharishi Dayanand University**

**Rohtak, (Haryana), India**

**Email: pundircs@rediffmail.com**

**Telephone no. 9416492413**

**Supplementary figure**

**
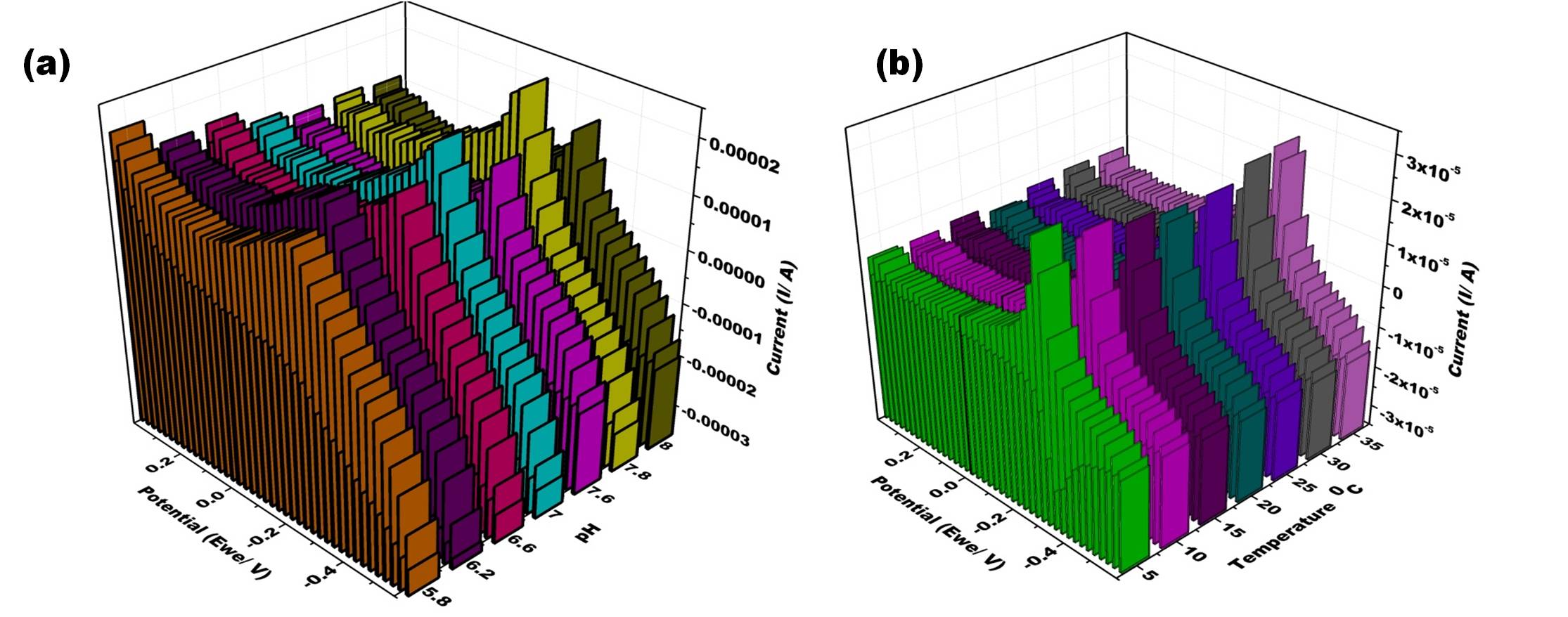
**

**Supplementary Fig. 1: (a) 3D representation of the cyclic voltammogram at PDNA/MoS_2_NSs/SPGE for pH of 0.1 M sodium phosphate buffer ranging from 5.8 to 8.0 each is having 1 µM MB in the potential range from -0.6 to + 0.4 V at the scan rate of 100 mVs^-1^. (b) 3D representation of the cyclic voltammogram at PDNA/MoS_2_NSs/SPGE for temperatures ranging from 5 to 35 ^0^C in 0.1 M sodium phosphate buffer ranging having 1 µM MB in the potential range from -0.6 to + 0.4 V at the scan rate of 100 mVs^-1^.**

**Supplementary Table 1: Comparison of MoS_2_ based sensors with the current sensor**

| **S. No.** | **Analyte** | **Detection technique** | **MoS_2_ or MoS_2_ based nanocomposites** | **Linear range** | **Limit of Detection** | **Reference** |
| --- | --- | --- | --- | --- | --- | --- |
|  | IgE | Electrochemiluminescence (ECL) | CdS–MoS_2_ nanocomposites | 0.001–10.0 nM | 0.34 pM | 37 |
|  | DNA | Electrochemical | MoS_2_ | 1.0 x 10^-16^ M to 1.0 x 10^-10^ M | 1.9 x 10^-17^ | 38 |
|  | DNA  RNA | Electrochemical | MoS_2_–thionin composite | 0.09 to 1.9 ngmL^-1^  10 to 200 ngmL^-1^ | 0.17 µA mL ng ^-1^  0.0022 µA mL ng ^-1^ | 39 |
|  | DNA | Electrochemical | MoS_2_/MWCNT | 10 to 10^7^ fM | 0.79 fM | 40 |
|  | IgE | Electrochemiluminescence (ECL) | CdSe/ZnS quantum dots (QDs)–MoS_2_ | 0.5 pM to 0.5 nM | 0.18 pM | 41 |
|  | ATP  Thrombin | Electrochemical | AuNPs–MoS_2_ nanocomposites |  | 0.74 nM  0.0012 nM | 42 |
|  | DNA | Electrochemica | MoS_2_ nanoflakes |  |  | 43 |
|  | DNA | Electrochemical | MoS2/graphene composites | 10^−16^ M to 10^−13^ M |  | 44 |
|  | Mercury Ion | Field-Effect Transistor | DNA functionalized Molybdenum Disulfide Nanosheet/Gold Nanoparticle Hybrid |  | 0.1 nM | 45 |
|  | DNA | Optical fibre based surface plasmon resonance (SPR) | MoS_2_-Graphene hybrid |  | 105.71deg/RIU | 46 |
|  | DNA | Capacitive displacement sensor | MoS_2_ nanoribbon |  | ∼70 million bases per second | 47 |
| 1. \ | DNA | Fluorescent Assay | MoS_2_/MWCNT nanocomposite | 0 – 50 nM | 1 nM | 48 |
|  | DNA | Photoluminescence | Au-Modified Monolayer MoS_2_ |  |  | 49 |
|  | Doxorubicin | Field effect transistors (FETs) | Cu^2+^ - DNA/MoS_2_ hybrid |  | 1.7 × 10^3^ A/A | 50 |
|  | Aptamer | Field effect transistors (FETs) | MoS_2_ nanosheet |  | 100 nM | 51 |
|  | Collagen peptide | Fluorescence | MoS_2_ | 25–300 nM | 41 nM | 52 |
